# Oxidation-Induced Increase In Photoreactivity of Bovine Retinal Lipid Extract

**DOI:** 10.1007/s12013-017-0832-3

**Published:** 2017-11-02

**Authors:** A. Koscielniak, M. Serafin, M. Duda, T. Oles, A. Zadlo, A. Broniec, O. Berdeaux, S. Gregoire, L. Bretillon, T. Sarna, A. Pawlak

**Affiliations:** 1 Department of Biophysics, Faculty of Biochemistry, Biophysics and Biotechnology, Jagiellonian University, Krakow, Poland; 2 Faculty of Electrical Engineering, Automatics, Computer Science and Biomedical Engineering, AGH-University of Science and Technology, Kraków, Poland; 3 INRA, Centre des Sciences du Gout et de l’Alimentation, Universite de Bourgogne, Dijon, France

**Keywords:** Retina, Lipids, Polyunsaturated fatty acids, Oxidation, Photor

## Abstract

The mammalian retina contains a high level of polyunsaturated fatty acids, including docosahexaenoic acid (22:6) (DHA), which are highly susceptible to oxidation. It has been shown that one of the products of DHA oxidation—carboxyethylpyrrole (CEP), generated in situ, causes modifications of retinal proteins and induces inflammation response in the outer retina. These contributing factors may play a role in the development of age-related macular degeneration (AMD). It is also possible that some of the lipid oxidation products are photoreactive, and upon irradiation with blue light may generate reactive oxygen species. Therefore, in this work we analysed oxidation-induced changes in photoreactivity of lipids extracted from bovine neural retinas. Lipid composition of bovine neural retinas closely resembles that of human retinas making the bovine tissue a convenient model for studying the photoreactivity and potential phototoxicity of oxidized human retinal lipids. Lipid composition of bovine neural retinas Folch’ extracts (BRex) was determined by gas chromatography (GC) and liquid chromatography coupled to an electrospray ionization source-mass spectrometer (LC-ESI-MS) analysis. Liposomes prepared from BRex, equilibrated with air, were oxidized in the dark at 37 °C for up to 400 h. The photoreactivity of BRex at different stages of oxidation was studied by EPR-oximetry and EPR-spin trapping. Photogeneration of singlet oxygen (^1^O_2_, ^1^Δ_g_) by BRex was measured using time-resolved detection of the characteristic phosphorescence at 1270 nm. To establish contribution of lipid components to the analysed photoreactivity of Folch’ extract of bovine retinas, a mixture of selected synthetic lipids in percent by weight (w/w %) ratio resembling that of the BRex has been also studied. Folch’s extraction of bovine neural retinas was very susceptible to oxidation despite the presence of powerful endogenous antioxidants such as *α*-tocopherol and zeaxanthin. Non-oxidized and oxidized BRex photogenerated singlet oxygen with moderate quantum yield. Blue-light induced generation of superoxide anion by Folch’ extract of bovine neural retinas strongly depended on the oxidation time. The observed photoreactivity of the studied extract gradually increased during its in vitro oxidation.

## Introduction

Retina, being a part of a central nervous system, shares its unique lipid composition [1–3]. Thus, up to 40% of phospholipids present in the retina contain polyunsaturated fatty acids (PUFAs) esterified mainly in the SN-2 position, and, to much lesser extent, also in the SN-1 position [4–7]. The most abundant PUFAs in the outer retina, especially in photoreceptor outer segments (POS), are docosahexaenoic acid (DHA (22:6)) and arachidonic acid (ARA (20:4)), which account for 21 and 10% of total fatty acids residues, respectively [1, 4, 8]. High concentration of PUFAs in POS is essential for maintaining the appropriate fluidity of their membranes, which is necessary for efficient visual transduction [9, 10]. However, high level of unsaturation makes PUFAs susceptible to oxidation. The outer retina, being exposed to intense irradiation from focused light, high oxygen concentration [11] and the presence of endogenous sensitizers, such as rhodopsin photobleached products [12, 13] or age pigment—lipofuscin [14, 15], is at elevated risk of oxidative stress.

Lipid peroxidation products are considered as an important factor causing irreversible modifications of cellular components, which in the long run may lead to the onset of degenerative processes, including age-related macular degeneration (AMD) [16]. It has been shown that 4-hydroxynonenal (4-HNE) or 4-hydroxyhexenal (4-HHE) [17, 18] and carboxyethyl pyrrole (CEP)—products of peroxidation of arachidonic and docosahexaenoic acids respectively, bind to cellular proteins forming advanced lipooxidation end-products (ALEs) [19, 20]. ALEs may induce an inflammatory response, which is supposed to play a role in the pathogenesis of AMD. The presence of CEP protein adducts in the outer retina are considered to be an early marker of high risk of AMD development [16, 21, 22].

Even though possible consequences of oxidation of retinal lipids for the onset and development of AMD were discussed, their potential photoreactivity and possible phototoxicity have not been considered. This is an important issue, considering that reactive products of lipid oxidation present in the outer retina, may be exposed to short-wavelength visible radiation [23–25]. If photoreactive, peroxidised retinal lipids may act as acquired endogenous sensitizers increasing the risk of retina photodamage. In this work, we analysed photoreactivity of Folch’ extract of bovine neural retinas (BRex) and oxidation-induced changes in its potential photoreactivity in heterogeneous model systems and in homogenous solutions. Since lipid composition of bovine neural retinas closely resembles that of human neural retinas, the bovine tissue becomes a convenient model for studying the postulated photoreactivity of human retinal lipids.

To establish contribution of lipid components to the analysed photoreactivity of Folch’ extract of bovine retinas, a mixture of selected synthetic lipids in percent by weight (w/w %) ratio resembling that of the BRex has been also studied. Photoreactivity of samples collected at selected oxidation times was analysed in liposomes and in their Folch’ extracts.

## Material and Methods

Chemicals, at least reagent grade, unless otherwise stated, were purchased from Sigma-Aldrich Inc. (All *trans* Retinal (atRAL), 5,5-dimethyl-1-pyrroline-*N*-oxide [DMPO], 5,10, 15,20-tetraphenyl-21H,23H-porphine [TPP], α-tocopherol (α-TOH), chelex), Avantor Performance Materials Poland (sodium phosphate, potassium phosphate, sodium chloride, potassium chloride, liquid chromatography grade benzene, chloroform, methanol) and used as supplied.

Cholesterol, Sphingomyelin (Brain, Porcine), 1-palmitoyl-2-docosahexaenoyl-*sn*-glycero-3-phosphatidylethanolamine (PE 16:0/22:6), 1-stearoyl-2-arachidonoyl-*sn*-glycero-3-phosphoethanolamine (PE 18:0/20:4), 1-palmitoyl-2-oleoyl-*sn*-glycero-3-phosphoethanolamine (PE 16:0/ 18:1), 1-palmitoyl-2-docosahexaenoyl-*sn*-glycero-3-phosphatidylcholine (PC 16:0/ 22:6), 1-stearoyl-2-arachidonoyl-*sn*-glycero-3-phosphocholine (PC 18:0/20:4), 1-palmitoyl-2-oleoyl-*sn*-glycero-3-phosphocholine (PC 16:0/18:1), 1,2-stearoyl-*sn*-glycero-phosphatidylcholine (PC 18:0/18:0) were purchased from Avanti Polar Lipids, Inc.

Bovine eyeballs were obtained from the local abattoir and transported to the laboratory on ice. The following procedure of bovine neural retinas collection was performed at dim light at 4 °C. Briefly: intact bovine eye globes were hemisected by an incision around the *pars plana*, the anterior segments (i.e., cornea, lens, vitreous) were removed and the neural retina was gently peeled and cut off from the optical nerve.

### Extraction of Lipids from BRex

Lipids and other hydrophobic components of bovine neural retinas were extracted from the tissue following slightly modified Folch’ method [26]. Shortly: collected bovine retinas were homogenized in a small amount of PBS (20 mM) using glass/PTFE manual homogenizer. Then suspension of homogenized tissue was mixed with Folch’ extraction mixture (chloroform: methanol, 2:1, v/v) in 5:8 (v/v) ratio and vortexed vigorously for a few minutes. Sample was centrifuged (15,000 rpm, 5 min, at 15 °C) and the chloroform layer was collected. To determine a dry mass of the obtained extract, chloroform was dried under a stream of argon and traces of organic solvent were removed by drying the sample under reduced pressure for 2 h. Obtained extract was stored under inert conditions and used for further experiments.

Phospholipid composition of collected Folch’ extracts bovine neural retinas, limited to phosphatidylcholines (PC) and phosphatidylethanolamines (PE), were analyzed using liquid chromatography coupled to an electrospray ionization source-mass spectrometer (LC–ESI–MS) as described elsewhere [4, 5], while free and phospholipid-esterified fatty acids after conversion to methyl esters using boron trifluoride (BF_3_) in methanol (7% w/v) were analysed with GC as previously described [27, 28].

### Detection of α-Tocopherol in BRex

The presence of α-tocopherol, endogenous, hydrophobic antioxidant in BRex was confirmed using HPLC-DAD Shimadzu at 290 nm. Separation was performed on C18 column (Beckman-ODS, 15 cm) with a mobile phase composed of mixture of methanol:acetonitrile:water:isopropanol (78:11:5.5:7.5; v/v) with the flow rate 1 ml/min. Pure α-TOH (12.5 mM) as a standard and freshly prepared BRex (2 mg/ml) were dissolved in a small amount of methanol and the injection volume was 10 μl. Retention time of α-TOH at the parameters used was 16 min.

### A Mixture of Synthetic Lipids

According to the BRex lipid composition determined by LC/MS and gas chromatography (GC) methods, a mixture of selected synthetic lipids, modelling those naturally present in the bovine neural retina and its Folch’ extract, were prepared. Such mixtures of lipids (25 mg/ml of total concentration) typically contained phospholipids (85 w/w %), cholesterol (10 w/w %) and sphingomyelin (5 w/w %). Detailed composition of this sample is given in Table 1.

**Table 1:** Composition of liposomes composed of selected synthetic lipids, naturally present in bovine neural retina

Lipids (w/w %)					
Cholesterol (10%)					
Sphingomyelin (5%)					
Phospholipids (85%)	PE (40%)	PE 16:0/22:6	SFAs (50 %)	MUFAs (12 %)	PUFAs (38 %)
		PE 18:0/20:4			
		PE 16:0/18:1			
	PC (60%)	PC 16:0/22:6			
		PC 18:0/20:4			
		PC 16:0/18:1			
		PC 18:0/18:0			

### Preparation of Multilamellar Liposomes

Multilamellar liposomes were prepared by the film deposition method as previously described [29–31]. Shortly, both Folch’ extract of bovine neural retinas obtained as described above and the mixture of selected synthetic lipids were dissolved in chloroform saturated with argon to prevent oxidation. Then, the chloroform was evaporated with a stream of argon or nitrogen gas, and the lipid film formed on the bottom of the test tube was thoroughly dried under reduced pressure for 6–12 h. A PBS (10 mM, pH 7.4), previously incubated at least 24 h with chelex to remove a trace of transition metal ions, was added to the dried Folch extract of bovine retinas and to the mixture of synthetic lipids at the room temperature and vortexed vigorously to complete removal of lipid film from the test tubes. The final concentration of BRex or the mixture of synthetic lipids in the obtained suspension of multilamellar liposomes was 25 mg/ml.

All preparations were performed in darkness or under dim light and, where possible, under nitrogen or argon.

### Oxidation of Liposomes

Both, multilamellar liposomes prepared from BRex and liposomes prepared from synthetic lipids at concentration 25 mg/ml were placed in a glass tubes, in a water bath at 37 °C in the dark. At zero time of oxidation, a part of liposomes was collected for analysis as a non-oxidized sample (0 h of oxidation), while the rest of sample was oxidized up to 400 h in the equilibrium with air. Concentration of BRex and synthetic lipids in liposomes was kept at 25 mg/ml by controlling the volume of oxidizing sample and, if necessary, by filling up with redistilled water previously incubated with chelex. At the selected time points of oxidation, part of liposomes was collected for analysis. To perform measurements in homogenous solutions, liposomes collected at various time points of oxidation underwent Folch’ extraction again and dried extract mass was determined. In following experiments, studied samples were normalized to their dry mass.

### Direct Detection of Singlet Oxygen (^1^Δ_g_, ^1^O_2_*) Phosphorescence at 1270 nm

Before measurements, freshly prepared and oxidized BRex in liposomes underwent Folch’ extraction again (2nd Folch extraction) to obtain homogenous solution of BRex in benzene and to determine quantum yield of ^1^O_2_ generation. The 2nd Folch’ extracts of non-oxidized and oxidized BRex, dried under stream of nitrogen, were re-dissolved in benzene, placed in a quartz fluorescence cuvette (QA-1000; Hellma, Mullheim, Germany) and excited with light generated by an integrated nanosecond DSS Nd:YAG laser system equipped with a narrow bandwidth optical parametric oscillator (NT242–1k-SH/SFG; Ekspla,Vilnius, Lithuania), which delivered pulses at repetition rate 1 kHz, with energy up to several hundred microjoules in the visible region, and up to several tens of microjoules in the UVA–UVB region. Quantum yield of singlet oxygen (^1^O_2_*, ^1^Δ_g_), generation upon excitation with 360 and 410 nm was determined by a comparative method, employing atRAL and TPP as standards [15, 32, 33]. In these experiments, initial intensities of singlet oxygen phosphorescence in the studied samples and in standards excited with laser pulses of selected wavelength were measured at increasing laser energies. Absorbance of the samples and standards was adjusted to ~ 0.10 at the excitation wavelengths (360 or 410 nm). Folch’ extracts of non-oxidized and oxidized liposomes composed of synthetic lipids were treated as described above. Quantum yield of singlet oxygen (^1^O_2_*, ^1^Δ_g_) generation was determined upon excitation with 360 nm only.

The near-infrared luminescence was measured perpendicularly to the excitation beam in a photon-counting mode using a thermoelectric cooled NIR PMT module (H10330-45; Hamamatsu, Japan) equipped with a 1100 nm cut-off filter and an additional dichroic narrow band filter NBP, selectable from the spectral range 1150–1355 nm (NDC Infrared Engineering Ltd, Bates Road, Maldon, Essex, UK). Data were collected using a computer-mounted PCI board multichannel scaler (NanoHarp 250; PicoQuant GmbH, Berlin, Germany). Data analysis, including first-order luminescence decay fitted by the Levenberg–Marquardt algorithm, was performed by custom-written software.

### Electron Paramagnetic Resonance (EPR)-Oximetry and EPR-Spin Trapping

To monitor visible light-induced consumption of oxygen in liposomes prepared from Folch extracts of bovine neural retinas, electron paramagnetic resonance (EPR) oximetry with the mHCTPO (0.1 mM) spin probe was employed. Measurements were carried out during in situ irradiation of the samples, placed in the resonant cavity as previously described [14, 34]. Visible light (395–700 nm, 34–40 mW/cm^2^) was derived from a 300 W high pressure compact arc xenon lamp (Cermax, PE300CE-13FM/Module300W; PerkinElmer Optoelectronics, GmbH, Wiesbaden, Germany) equipped with a water filter, heat reflecting hot mirror and cutoff filter blocking light below 395 nm. In case of sample irradiation with blue light, blue additive dichroic filter 505FD64–25 (Andover Corporation, Salem, NC, USA) was also used.

EPR measurements were performed using the following instrument parameters: microwave power 1.06 mW, modulation amplitude 0.006 mT, scan width 0.3 mT, and scan time 5.2 s using a Bruker EMX-AA 1579 EPR spectrometer (Bruker BioSpinGermany).

EPR spin trapping measurements were performed using DMPO (0.1 M, H_2_O) as a spin trap. Samples containing non-oxidized or oxidized BRex (1.3 mg/ml) in a mixture containing benzene:DMSO:H_2_O (1:8:1, v/v/v) were irradiated with blue light (404–515 nm, 50–58 mW/cm^2^), employing the same light source and filters as those described above. Time-dependent photo-accumulation of the DMPO-OOH spin adducts was measured.

EPR spin-trapping measurements were performed at employing the following parameters: microwave power 10.6 mW, modulation amplitude 0.05 mT, scan width 8 mT, and scan time 84 s.

## Results

### Detection of α-TOH

**Fig. 1:**
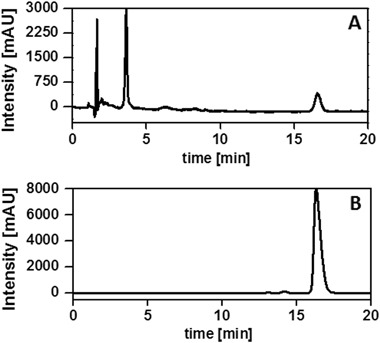
Representative HPLC chromatograms of α-tocopherol in: Folch extract of bovine retinas **a** and solution of measured amount of α-tocopherol used as a standard **b**. Sample absorbance was measured at 290 nm and the, retention time for α-tocopherol was 16 min

Bovine neural retinas Folch’ extract, besides lipids, also contained hydrophobic antioxidants such as α-tocopherol and zeaxanthin. Representative chromatograms at 295 nm of non-oxidized BRex and α-TOH used as a standard, are shown in Fig. 1 Retention time of α-TOH was about 16 min, consistent with literature data [35]. The level of α-tocopherol (1.5–3 μM) in BRex is comparable to that reported by Dilley and McConnell [36] and reaches 0.1% mol relative to phospholipids [36, 37]. The presence of zeaxathin in BRex was confirmed using Raman Confocal Microscopy (data not shown).

### Lipid Composition Analysis

Individual fatty acids identified in freshly prepared BRex, shown in Table 2, are grouped as saturated (SFAs), monounsaturated (MUFAs) and PUFAs. The predominant fatty acids of the SFAs group are palmitic (16:0) and stearic (18:0) acids, which constituted up to 40% of all fatty acids in the examined extract. MUFAs is represented by oleic acid (18:1, n-9), which is also the most abundant fatty acid of its group reaching over 70% of all MUFAs. Nearly 40% of all fatty acids residues in BRex are PUFAs with DHA (22:6) and ARA (20:4), which account for 22.9 and 8.7% of total FA residues in the bovine neural retina, respectively.

**Table 2:** Fatty acids composition of total lipids extracted from bovine neural retinas by the Folch method determined by gas chromatography

Fatty acids	BRex	Main fatty acids
		(% of all FA in BRex)
SFA [%]	41.03 ± 3.07	**16:0** (20.29 ± 1.02)
		**18:0** (19.46 ± 2.00)
MUFA [%]	13.67 ± 0.80	**18:1n-9** (10.23 ± 0.76)
PUFA [%]	39.88 ± 3.78	**22:6n-3** (22.93 ± 2.69)
		**20:4n-6** (8.74 ± 0.49)

Mean content (in % of all identified fatty acids) of SFA, MUFA, and PUFA as well as the most abundant representatives of each group of fatty acids in the studied extract are presented

Phospholipids constitute 78% of a dry mass of the extract (according to phosphorus content detected, data not shown). The most abundant phospholipids in BRex are phosphatidylcholines and phosphatidylethanolamines and the primary components of both groups are presented in Tables 3 and 4, respectively. Most of SFAs are esterified in phosphatidylcholines, while PUFAs in phosphatidylethanolamines (Tables 3 and 4).

**Table 3:** Most abundant choline-phospholipids (in % of all phosphatidylcholines) in bovine retinas Folch’s extract identified by liquid chromatography coupled with tandem mass spectrometry

Molecular species of phosphatidylcholine	% of all PC identified in BRex
PC 16:0/16:0 (DPPC)	14.34 ± 1.54
PC 16:0/18:1 (POPC)	19.55 ± 0.09
PC 16:0/20:4 and/or PC 18:2/18:2	4.19 ± 0.69
PC 16:0/22:6 and/or PC 18:2/20:4	6.44 ± 0.38
PC 18:0/22:6 and/or PC 20:2/20:4	10.94 ± 0.84
PC 16:0/18:0 (PSPC)	5.05 ± 0.14

**Table 4:** Most abundant ethanolamine-phospholipids (in % of all phosphatidylethanolamines) in bovine retinal Folch’s extract identified by liquid chromatography coupled with tandem mass spectrometry

Molecular species of phosphatidylethanolamine	% of all PE identified in BRex
PE 16:0/18:1 (POPE)	2.39 ± 1.37
PE 18:0/18:1 (SOPE)	2.06 ± 0.71
PE 18:0/20:4	7.98 ± 0.96
PE 18:0/22:6	38.18 ± 0.29
PE 18:1/22:6	4.13 ± 0.26
PE 22:6/22:6	1.94 ± 0.06

### Photo-Induced Oxygen Uptake

**Fig. 2:**
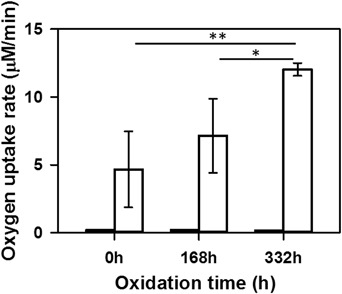
Oxygen consumption in liposomal samples containing Folch extracts of bovine retinas subjected to selected oxidation times. Black and white bars represent dark and light-induced (395–700 nm, 34–40 mW/cm^2^) initial rates of oxygen uptake (μM/min), respectively. Increase in oxygen photo-consumption rate observed after 332 h of oxidation of BRex in liposomes is statistically significant in comparison to non-oxidized (*P* < 0.005) and oxidized for 168 h samples (*P* < 0.02)

The rate of oxygen uptake in liposomes prepared from BRex depended on oxidation time and was apparently enhanced by irradiation of the samples (Fig. 2). Oxygen uptake rate monitored in all BRex containing liposomes in the dark was very low and did not exceed the value of 0.2 μM/min. However, in non-oxidized liposomes irradiated with visible light (395–700 nm, 34–40 mW/cm^2^) the oxygen consumption rate reached 4.7 μM/min and increased to 7 and 11 μM/min in samples oxidized for 168 and 332 h, respectively. The observed increase in oxygen photo-consumption rate in liposomes oxidized for 332 h compared to control (non-oxidized BRex) was statistically significant (*P* < 0.005). Also the difference in oxygen photoconsumption rates between liposomes oxidized for 168 h and those oxidized for 332 h was statistically significant (*P* < 0.02) (Fig. 2).

**Fig. 3:**
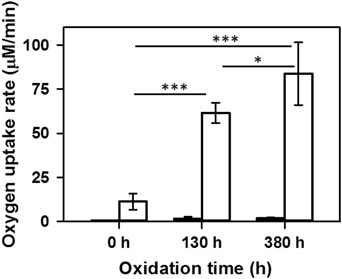
Oxygen consumption in liposomal samples composed of a mixture of synthetic lipids, naturally present in the bovine retinas, in ratio resembling that of Folch’ extract of bovine retinas, subjected to selected oxidation times. Black and white bars represent dark and blue light-induced (395–700 nm, 34–40 mW/cm^2^) initial rates of oxygen uptake (μM/min), respectively. Increase in oxygen photo-consumption rate observed after 130 and 380 h of oxidation of studied samples is statistically significant in comparison to non-oxidized sample (*P* < 0.001). Also, the difference in oxygen photo-uptake rate between samples oxidized 130 and 380 h is statistically significant (*P* < 0.02)

Results of experiments performed on the mixture of synthetic lipids, indicate that oxygen photo-uptake rate in non-oxidized sample reached 11 μM/min (Fig. 3) and was two times higher than that detected in non-oxidized BRex (Fig. 2). Moreover, although the rate of oxygen uptake in BRex slowly increased with oxidation time (Fig. 2), in liposomes composed of synthetic lipids and oxidized just 130 h, it increased more abruptly to a higher level (61 μM/min) (Fig. 3). These results confirm the role of hydrophobic endogenous antioxidants present in Folch’ extract of bovine retinas.

### Singlet Oxygen Generation

**Fig. 4:**
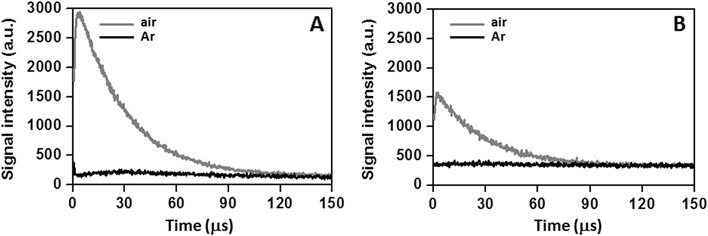
Time-resolved singlet oxygen (^1^Δ_g_, ^1^O_2_) phosphorescence at 1270 nm detected in samples of oxidized (168 h) Folch extract of bovine retinas upon excitation with 360 nm (**a**) and 410 nm (**b**) laser pulses. 1270 nm luminescence was acquired in air equilibrated (grey line) or argon-saturated (black line) samples dissolved in benzene

To determine the mechanism responsible for the observed oxygen uptake in irradiated samples, photogeneration of singlet oxygen by BRex in homogenous solution was studied. Non-oxidized and oxidized BRex for 168 and 332 h in liposomes were extracted again (the 2nd Folch extraction) and re-dissolved in benzene at concentration 2 mg/ml. Excitation of the studied extracts with laser pulses at 360 or 410 nm, induced phosphorescence, which decayed with time constant characteristic for singlet oxygen lifetime (Fig. 4). Disappearance of the observed infrared luminescence occurred after prolonged saturation of the examined samples with argon. Representative results acquired in air-equilibrated and Ar-saturated solution of oxidized (168 h) BRex excited with 360 or 410 nm are shown in Fig. 4a and b. Efficient reduction in lifetime of the detected phosphorescence at 1270 nm was observed in the presence of sodium azide (100 mM), a well-known singlet oxygen physical quencher (data not shown).

Using atRAL [15] and TPP [33] as singlet oxygen generation standards, quantum yield of ^1^O_2_ generation by non-oxidized and oxidized BRex was determined (Table 5). The determined quantum yield of ^1^O_2_ generation upon excitation at 360 and 410 nm was 0.08 and 0.06 in the case of BRex oxidized for 332 h, respectively. In turn, quantum yield of singlet oxygen generation by non-oxidized mixture of synthetic lipids, determined upon excitation with 360 nm reached 0.095 and slightly increased up to 0.13 after 130 h of oxidation (Table 6). However, after 380 h of oxidation, the quantum yield of singlet oxygen generation decreased to 0.11 following the same trend, which was observed in the case of BRex (Table 5).

**Table 5:** Quantum yield (Φ) of singlet oxygen (^1^O_2_, ^1^Δ_g_) generation by non-oxidized (0 h) and oxidized (168 h or 332 h) Folch’ extract of bovine retinas determined in homogenous benzene solution upon excitation with 360 nm or 410 nm laser pulse

Oxidation time	Quantum yield (Φ) of ^1^O_2_ generation in BRex upon excitation with:		Lifetime of ^1^O_2_ (μs)
	360 nm	410 nm	
0 h	0.088 ± 0.006	0.042 ± 0.005	22.3 ± 0.4 **
168 h	0.091 ± 0.025	0.050 ± 0.019	22.6 ± 0.2
332 h	0.083 ± 0.022	0.057 ± 0.019	24.2 ± 0.1 **
Standards: at RAL	0.30^a^	32.1 ± 0.4	
TPP	0.63^b^	30.1 ± 0.2	

Singlet oxygen generation standards used: all *trans* retinal (Φ = 0.3) and TPP (Φ = 0.63). The last column are lifetimes of singlet oxygen in the samples studied. Difference between singlet oxygen lifetime generated in non-oxidized BRex and BRex oxidized for 332 h is statistically significant (*P* < 0.01)

^a^ Rozanowska et al. (1998) [15]

^b^ Bonnett et al. (1988) [33]

** Significance at the 0.01 probability level

**Table 6:** Quantum yield (Φ) of singlet oxygen (^1^O_2_, ^1^Δ_g_) generation by non-oxidized (0 h) and oxidized (130 h or 380 h) Folch’ extract of liposomes composed of mixture of synthetic lipids in ratio resembling that of Folch’ extract of bovine retinas

Oxidation time	Quantum yield (Φ) of ^1^O_2_ generation in extract of liposomes composed of synthetic lipids naturally present in BRex upon excitation with:	Lifetime of ^1^O_2_ (μs)
	360 nm	
0 h	0.095 ± 0.020	28.6 ± 0.2
130 h	0.130 ± 0.005	27.6 ± 0.2
380 h	0.110 ± 0.004	27.0 ± 0.1
Standards: at RAL	0.30^a^	32.1 ± 0.4
TPP	0.63^b^	30.1 ± 0.2

Quantum yields were determined in homogenous benzene solution upon excitation with 360 nm laser pulse. Singlet oxygen generation standards used: all *trans* retinal (Φ = 0.3) and TPP (Φ = 0.63). The last column are lifetimes of singlet oxygen in the samples studied

^a^ Rozanowska et al. (1998) [15]

^b^ Bonnett et al. (1988) [33]

Although in the case of BRex, the quantum yield of singlet oxygen generation upon excitation with 360 nm seems to be independent on oxidation time, the amount of ^1^O_2_ generated by BRex upon excitation with 410 nm, slightly increased with the oxidation time (Table 5).

**Fig. 5:**
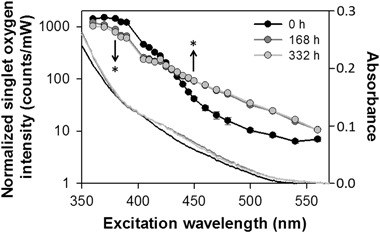
Action spectra of photo-generation of singlet oxygen by non-oxidized and oxidized for 168 and 332 h Folch’ extracts of bovine retinas in benzene. For comparison, the corresponding absorbance spectra of studied samples are also shown. Initial intensities of singlet oxygen phosphorescence at 1270 nm, normalized to equal laser power, are plotted as a function of excitation wavelength. Differences in initial intensities of singlet oxygen luminescence generated by non-oxidized and oxidized for 168 h bovine retinas Folch’ extracts are statistically significant as indicated in the plot for selected excitation wavelength (375 and 450 nm, *P* < 0.03 and *P* < 0.02, respectively)

Dependence of the initial intensity of ^1^O_2_ phosphorescence on excitation wavelength was examined for non-oxidized (0 h) and oxidized BRex for 168 and 332 h. The obtained action spectra in the spectral range the 360–550 nm are shown in Fig. 5 Interestingly, initial intensity of the detected luminescence strongly depended on excitation wavelength and differed significantly from the absorption spectra of the studied extracts (Fig. 4). Oxidation of the studied extract apparently reduced the intensity of ^1^O_2_ phosphorescence in the UV excitation range. However, an increased efficiency of singlet oxygen generation by oxidized BRex, in comparison with non-oxidized sample, was observed in the short-wavelength visible light region (Fig. 5).

The observed differences in initial intensities of singlet oxygen luminescence generated by non-oxidized and oxidized for 168 h bovine retinas Folch’ extracts are statistically significant as indicated in Fig. 5 for the excitation wavelength 375 and 450 nm, (*P* < 0.03 and *P* < 0.02, respectively).

### Generation of Free Radicals

**Fig. 6:**
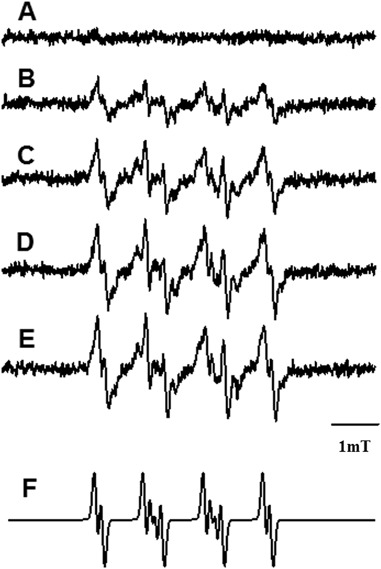
EPR-spin trapping of superoxide anion in irradiated sample of oxidized (398 h) Folch’ extract of bovine retinas. Sample, at the concentration 1.3 mg, was dissolved in a mixture of DMSO:benzene:water, (8:1:1, v/v/v). EPR spectra of the detected spin adduct were recorded in sample irradiated with blue light (404–515 nm, 50–58 mW/cm^2^) for 0 min (a), 6 min (b), 16 min (c), 23 min (d) and 30 min (e). Simulated spectrum of the DMPO-OOH spin adduct is shown in (f)

**Fig. 7:**
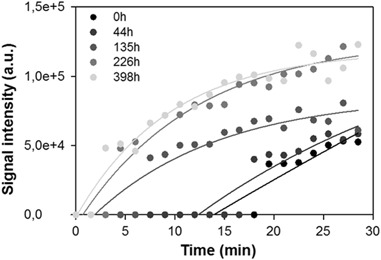
Time-dependent changes in the intensity of DMPO-OOH spin adduct during blue light irradiation of non-oxidized (0 h) and oxidized for 44, 135, 226 and 398 h Folch’ extract of bovine retina. Other experimental conditions as in Fig. 6

Using EPR spin-trapping method with DMPO as a spin trap, formation of a characteristic spin adduct was observed in samples containing non-oxidized and oxidized BRex irradiated with blue-light. The 2nd Folch extracts of non-oxidized and oxidized BRex in liposomes were re-dissolved in benzene and EPR spectra acquired at various time of illumination of oxidized for 398 h BRex are presented in Fig. 6. The hyperfine coupling constants (hsc) A_N_ = 12.9 G, A_H1_ = 10.71 G and A_H2_ = 1.27 G of the collected spectra indicate that the observed spin adduct is DMPO-OOH—a product of the interaction of DMPO with superoxide anion radical (O_2_^●−^). Indeed the reported hyperfine splitting constants arising from the superoxide anion adduct with DMPO are: A_N_ = 12.63 G, A_H1_ = 10.14 G and A_H2_ = 1.28 G [38]. A small amount of oxidized lipid-derived peroxyl radical spin adduct (DMPO-OOR) may also contribute to the detected EPR spectrum in sample of oxidized for 398 h BRex (Fig. 6) [39, 40]. Intensities of EPR spectra of the DMPO-OOH/DMPO-OOR spin adducts in all studied samples gradually increased with time of blue-light irradiation (Fig. 7).

## Discussion

The observed photoreactivity of non-oxidized Folch’ extract of bovine neural retinas may be attributed to the presence of endogenous sensitizers such as products of visual pigment bleaching, flavins or porphyrins [12, 41, 42]. These endogenous photosensitizers could be responsible for the relatively low oxygen consumption rate (5 μM/min) induced by visible light (Fig. 2). Very low rate of oxygen consumption (below 0.2 μM/min) was observed in the dark regardless of the degree of BRex oxidation.

The observable rate of oxygen photoconsumption may be affected by the presence of endogenous antioxidants. Even though the estimated level of α-TOH in BRex was quite low, it was comparable to the amount of α-TOH in the dark adapted bovine neural retina (42 ± 1 nmol/g of dry tissue), as previously reported [43], while in the rod outer segments (ROS) α-TOH accounted for about 1 nmole per 1 mg of protein [36]. Such an amount of α-TOH is roughly equivalent to 0.1 mol% in relation to phospholipids in bovine ROS [44]. It was also reported that the level of α-TOH in the monkey retina positively correlated with retinal PUFAs concentration and the age of donors [45]. Macular pigments (MP)—lutein and zeaxanthin, are also present in the bovine ROS [46], albeit in a very low amount (0.2–0.4 ng/mg of protein) [47], comparable to that found in human peripheral retina [48, 49].

The effect of endogenous antioxidant present in BRex was also apparent when generation of singlet oxygen was analysed (Table 5), particularly in comparison with that in extract of liposomes containing synthetic lipids only (Table 6). Quantum yields of ^1^O_2_ generation by non-oxidized BRex and by a mixture of synthetic lipids suggest that a minor contribution to the observed generation of singlet oxygen may be due to the presence of other than lipid components. However, it cannot be ruled out that in the case of synthetic lipids, some of its components, especially these phospholipids containing PUFA, were partially oxidized contributing to the UV-induced singlet oxygen generation, as it has been reported [50–52]. On the other hand, freshly prepared BRex may contain small amounts of all-trans retinal, which would contribute to photogeneration of ^1^O_2_ by BRex. It is apparent that the presence of endogenous antioxidants in BRex reduces the amount of the photogenerated singlet oxygen and its lifetime by several percent in both non-oxidized and oxidized samples.

In benzene, the lifetime of singlet oxygen generated by TPP and atRAL was found to be 32.1 ± 0.4 and 30.1 ± 0.2 μs, respectively, in agreement with reported data [53, 54]. However, the lifetime of singlet oxygen was significantly shorter, if it was photogenerated, under the same experimental conditions, by non-oxidized (22.3 ± 0.4 μs) and oxidized for 168 h and 332 h BRex (22.6 ± 0.2 and 24.2 ± 0.1 μs, respectively). Difference between singlet oxygen lifetime generated in non-oxidized BRex and BRex oxidized for 332 h is statistically significant (*P* < 0.01). Such a shortening of singlet oxygen lifetime was not observed when ^1^O_2_ was photo-generated in extract of non-oxidized (28.6 ± 0.2 μs) and oxidized for 130 h and 380 h liposomes containing only synthetic lipids (27.6 ± 0.2 and 27.0 ± 0.1 μs, respectively) (Table 6). Even though the main component of the examined extract were phospholipids PL (78%), at the BRex concentration used, the estimated effect of phospholipids on ^1^O_2_ lifetime would be too small to significantly shorten the observable singlet oxygen lifetime, consistent with the results observed in samples containing only synthetic lipids. Assuming that the average rate constant of PL interaction with ^1^O_2_ is 1 × 10^5^ M^−1^s^−1^ [55], the expected shortening of singlet oxygen lifetime should not exceed several percent. Although the reported rate constant of ^1^O_2_ quenching by α-TOH is 2 × 10^8^ M^−1^s^−1^ [56], concentration of this antioxidant in BRex was too low to have an impact on the observable singlet oxygen lifetime. On the other hand, macular pigments (MP): zeaxanthin and lutein, interact with ^1^O_2_ with much higher rate constants being 1.26 × 10^10^ M^−1^s^−1^ and 1.70 × 10^10^ M^−1^s^−1^ respectively [57]. Thus, even at a low concentration MP in BRex, such as 1 μM, they could reduce the observable lifetime of ^1^O_2_ by approximately 30%. Of course, presence of any other compounds that are also able to interact with ^1^O_2_ would also contribute to the observable effect.

Interestingly, long-term (332 h) oxidation of BRex increased the detected lifetime of singlet oxygen by 10% (Table 5). It may suggests that concentration of possible quenchers of ^1^O_2_ was reduced by prolonged oxidation of the extract. Although the predominant mode of singlet oxygen quenching by xanthophylls and tocopherols is physical, singlet oxygen can also induce oxidation of these antioxidants [43, 48]. Indeed, Khachik et al reported not only the presence of lutein and zeaxanthin in the human retina but also their oxidation products [47].

The main aim of this study was to analyse the postulated photoreactivity of retinal lipids and to determine if the photoreactivity is modulated by oxidation of the lipids. The Folch’ extraction is regarded as one of the most reliable methods for isolation of a broad range of lipid classes from biological materials [58, 59]. The predominant component of the Folch’ extract of bovine retinas are lipids, which were analysed in this study. Phospholipids, mainly phosphatidylcholines and phosphatidylethanolamines, constituted 78% of the extract dry mass. Phosphatidylserines, which usually account for less than 10% of PL in the vertebrate retinas, for technical reasons, were not analysed in this study [1]. Detailed analysis of fatty acids esterified in the main class of phospholipids studied, i.e., PC and PE revealed that most of saturated FA were esterified in PC, while polyunsaturated FA in PE (Tables 3 and 4), which is in agreement with previously published data [6, 60, 61].

It must be emphasized that the fatty acids and phospholipid composition of the bovine neural retinas is quite similar to that of the human neural retina [1, 47, 61], which makes bovine retinas a convenient model system of the human retina when analysing processes leading to oxidative modifications of the retinal lipids.

The rate of oxygen consumption induced by irradiation with short-wavelength visible light was significantly higher in oxidized BRex, compared to non-oxidized samples (Fig. 2). Also, the amount of ^1^O_2_ generated by BRex upon excitation with 410 nm, slightly increased with the oxidation time (Table 5) and the difference in initial intensities of singlet oxygen luminescence generated by non-oxidized and oxidized for 168 h BRex is statistically significant at 450 nm excitation wavelength (*P* < 0.02) (Fig. 5).

The higher rate of oxygen photoconsumption in such samples was accompanied by their increased ability to generate superoxide radical (Fig. 7).

The proper function and well-being of the retina depends on efficient interactions between photoreceptor cells, responsible for collecting and processing visual stimuli, and retinal pigment epithelium cells (RPE), which plays an important supporting role and is involved in biological renewal of photoreceptor outer segments [62]. Both types of the retina cells are characterized by high lipid metabolism [63, 64]. Discs containing visual pigment, continuously formed in photoreceptor inner segments, are directed to POS, where they form stacks in rods or series of invaginations in the case of cone photoreceptor cells [63, 65]. POS maintain a roughly constant length by exfoliating mature discs from the top, which in turn are subsequently phagocytosed by adjacent RPE cells [66]. Photoreceptor renewal is one of the most efficient mechanism, which prevents the retina from degeneration [67]. POS plasma membrane and POS discs membranes are especially rich in lipids containing PUFAs [68, 69]. This unique lipid composition, localization, and role they play in the outer retina, increase the risk of oxidative stress. Disks undergo shedding and phagocytosis 9–12 days after assembling and subsequent displacement in direction to the top of POS [66, 70]. It is probable that lipids present in discs, being phagocytized, are oxidized to some extent [71, 72].

The detection methods and characterization of the nature and biological role of oxidation products of different class phospholipids have been amply described in numerous papers [73–76]. Lipid oxidation products are commonly consider as an efficient protein modification factor and/or signalling molecules involved in various processes [77–79], also occurring in the retina [23, 80–83]. However, to our best knowledge, none of these reports discussed the significance of potential photoreactivity of lipid oxidation products, upon irradiation with visible light in the retina, where they might serve as potential photosensitizers.

Age-related decrease in the efficiency of RPE cells to digest phagocytised POS can lead to elevated accumulation of partly degraded and undigested material in secondary lysosomes in RPE cells [84, 85]. It must be stressed that the increasing photoreactivity of human RPE cells that occur with senescence can also result from accumulation of the age pigment lipofuscin, which in model systems exhibited substantial photoreactivity and was shown to be phototoxic in RPE cells in vitro [15, 86–88]

## Conclusion

Freshly prepared bovine retinas Folch extract exhibited moderate photoreactivity, when irradiated with blue light, and the photoreactivity increased as a result of oxidation carried out under mild conditions.

In vitro oxidation of liposomes prepared from BRex induced broadening of absorbance spectra and appearance of blue light absorbing components.
